# Heart weight must not be measured before dissection during autopsies

**DOI:** 10.1007/s00414-023-03089-9

**Published:** 2023-09-18

**Authors:** Larissa Lohner, Christoph Sinning, Anna Isabella Suling, Rexson Tse, Jack Garland, Benjamin Ondruschka

**Affiliations:** 1 Institute of Legal Medicine, University Medical Center Hamburg-Eppendorf, Hamburg, Germany; 2 University Heart Center, University Medical Center Hamburg-Eppendorf, Hamburg, Germany; 3 Institute of Medical Biometry and Epidemiology, University Medical Center Hamburg-Eppendorf, Hamburg, Germany; 4 Griffith University School of Medicine, Southport, QLD Australia; 5 Queensland Public Health and Scientific Services, Coopers Plains, QLD Australia

**Keywords:** Forensic autopsy, Heart weight, Sudden cardiac death, Cardiomegaly, Cardiac hypertrophy

## Abstract

During autopsies, weighing the heart is a standard procedure. In addition to myocardial pathologies, heart size, and ventricular wall thickness, heart weight is a common parameter to describe cardiac pathology and should be recorded as accurately as possible. To date, there exists no standard for recording heart weight at autopsy, although some authors recommend weighing the heart after dissection and removal of blood and blood clots. In the study presented, the hearts of 58 decedents were weighed after being dissected out of the pericardial sac (a), after dissection using the short-axis or inflow-outflow method with manual removal of blood and blood clots (b), and after rinsing and drying (c). Depending on the dissection method, the heart weight was 7.8% lower for the inflow-outflow method and 11.6% lower for the short-axis method after dissection compared to before and correspondingly 2.9% to 5% lower again after rinsing and drying respectively. Accordingly, the heart should be dissected, blood and blood clots removed, rinsed with water, and dried with a surgical towel after dissection, before weighing.

## Introduction

Heart weight is an important parameter in pathological and forensic autopsy practice and should be recorded as accurately as possible since cardiovascular diseases are the leading causes of death worldwide [1]. Sudden cardiac death (SCD) remains one of the most common causes of mortality and is currently reported as the third leading cause of death [2, 3]. Sudden cardiac death is defined as unexpected, nontraumatic death occurring within one hour of the onset of new or worsening symptoms (witnessed cardiac arrest) or, if unwitnessed, within 24 h of last being seen alive [4]. With this in mind, the diagnosis of SCD is most commonly made for individuals who were previously considered healthy or who had underlying disease but the sudden deterioration resulting in death was unexpected [5]. In forensic pathology, the most common parameters for objective description of cardiac pathology, in addition to morphology of myocardial pathologies, are the measurement of ventricular wall thicknesses and recording of total heart weight during autopsy [5, 6]. Increased heart weight is a significant and independent factor associated with SCD, despite being a poor predictor for it [7]. According to the "Guidelines for autopsy investigation of sudden cardiac death" [5, 8, 9], the heart should be prepared using the short-axis method and weighed after removal of blood and blood clots. However, an exact time point is not specified.

To the best of our knowledge, no standardized method exists at the present time for recording total heart weight. In a monocentric study by Garland et al. [10], the total heart weight was determined at two different measurement time points (after dissection and rinsing and after pat drying of the heart). The heart was dissected using the short-axis or inflow-outflow method. Heart weight was significantly lower after pat drying. No statistically significant differences were found between the dissection methods. Loper et al. [11] published a study in which total heart weight was determined at three time points during autopsy (after dissecting out of the pericardial sac, after removal of blood without dissection of the heart, and after complete short-axis dissection with removal of blood and drying of the heart). This study also showed significant differences in heart weights at the three different time points with the lowest weight after dissection and pat drying.

The aim of the present study was to review previously published studies, and to recommend an optimal time point for weighing the heart in autopsy practice as well as to investigate a possible difference between the dissection methods. Heart weight was determined at three different time points: (a) after dissecting the heart out of the pericardial sac, (b) after complete dissection using the inflow-outflow or the short-axis method with manual removal of blood and blood clots, (c) after rinsing and drying.

## Material and methods

The study was conducted at the Institute of Legal Medicine in Hamburg, Germany, from 01/01/2022 to 12/31/2022. Decedents over 18 years of age who had echocardiography up to six months before death were included for investigation as part of a higher-level study. Exclusion criteria were decedents with an artificial or biological heart valve replacement, ruptured myocardial infarctions, recent cardiac surgery, and after ECMO (Extracorporal membrane oxygenation) insertion, putrefaction, and polytrauma. The study protocol was approved by the Ethics Committee of the Hamburg Medical Association (Application Number 2020–10311-BO-ff). The closest relatives were contacted by telephone for the examination and informed about the study.

After consent by the closest relatives, a partial autopsy was performed with examination of the thoracic cavity, and evisceration of the heart from the pericardial sac. The aorta and pulmonary vessels were cut 2 cm above the semilunar valve. Dissection was performed according to European and American guidelines [5, 12] using either the inflow-outflow (*n* = 30) or short-axis (*n* = 28) method allocated in a random manner by the same forensic pathologist (first author). In the inflow-outflow method, the right atrium and ventricle were opened and the pulmonary outflow tract was dissected according to the direction of blood flow. Then, the left atrium and left ventricle were opened toward the apex of the heart and back across the aorta. In the short-axis method, a transverse incision was made at the level of the mid ventricle with further parallel transverse slices at 1 cm intervals towards the apex. The remainder of the right and left ventricles in the basal half of the heart were dissected in the direction of blood flow.

In total, the heart weight, including epicardial fat, of 58 decedents was determined at three measurement time points using the bench scale KERN FCF 30 K-3 (Kern und Sohn GmbH Balingen, Germany) with a capacity of 30 kg and an error margin of 1 g. The first measurement was performed after dissecting the heart out of the pericardial sac without removal of blood, postmortem and blood clots (a). After dissection using the inflow-outflow or the short-axis method, blood, postmortem clots and blood clots were removed manually (b). After rinsing and drying the heart by applying gentle pressure using a surgical towel, the third measurement was performed (c). In addition, sex, age, postmortem interval (PMI), body length and weight, body mass index (BMI), previous diseases, and clinical cause of death were also recorded.

The α-level was set to 5%. Continuous variables were described using mean, median, standard deviation (SD), and range (minimum—maximum). A linear mixed model was used to determine differences in heart weight between the three time points and between dissection methods. Change in heart weight from baseline (after dissecting out of the pericardial sac) represented the dependent variable. Decendents ID was included as a random factor and the model was adjusted for sex, age, BMI, and baseline heart weight. The interaction between time and dissection method was also tested in the model. Results are presented as adjusted means together with 95% confidence intervals (CI) and a descriptive percentual change calculated as change from the mean baseline value.

Statistical analysis was performed using IBM SPSS Statistics version 29.0 (IBM, Armonk, New York, USA) and Stata 17.0 (StataCorp. 2021. Stata Statistical Software: Release 17. College Station, TX: StataCorp LLC).

## Results

Among the 58 cases, the sex ratio was balanced (female *n* = 29, male *n* = 29) with a mean age of 72.2 years (SD 16.3, median: 77 years, range: 29—100 years). The mean body length was 170.1 cm (SD 12.2, median: 169.5), body weight 76.7 kg (SD 25.6, median: 68.2) with a mean BMI of 26.2 kg/m^2^ (SD 6.8, median: 24.3 kg/m^2^). The inflow-outflow method was used in 30 cases and the short-axis method in 28 cases. The postmortem interval averaged 2.9 days (SD 2.4, median: 2.1, range: 0.4—14.6). After the delivery to the Institute of Legal Medicine Hamburg, Germany, all bodies were cooled at 4 degrees Celsius until they were examined. The most common clinically described causes of death were cardiovascular (*n* = 14) and carcinoma (*n* = 12), followed by pneumonia (*n* = 11), sepsis (*n* = 6), gastrointestinal (*n* = 5), and multiple organ failure (*n* = 5), respiratory failure (*n* = 2), and stroke (*n* = 1). In one individual, the cause of death was clinically unclear.

### Comparison of the heart weights of the individual measurement time points without dissection method comparison

After dissecting the heart out of the pericardial sac (a), the mean heart weight was 558.8 g (SD 199.7, median: 518.0, range: 267.0 – 1121.0). In men, the average heart weight was 623.3 g (SD 203.9, median: 575.0, range: 360–1121), and in women 494.2 g (SD 176.0, median: 480.0, range: 267.0–925.0). After dissection and manual removal of blood, blood clots, and postmortem clots (b), the mean heart weight was 504.3 g with a median of 451.0 g (SD 178.1, range: 242.0–1026.0) (men: 568.9, SD 173.8, median: 510.0, range: 337.0–1026.0; women: 439.8, SD 160.3, median: 407.0, range: 242.0–840.0). After rinsing and drying (c), the heart weight was 484.6 g (SD 168.0, median: 437.5, range: 210.0–1016.0). For men, the average heart weight after rinsing and drying was 550.7 g (SD 165.9, median: 507.0, range: 334.0–1016.0), and for women 418.4 g with a median of 389.0 g (SD 144.4, 210.0–739.0).

There was a weight difference of 54.4 g (SD 51.9, median: 30.5) (9.7%) between measurement time points (b) and (a), and a difference of 74.2 g (SD 59.8, median: 56.0) (13.3%) between measurement time points (c) and (a). The difference between measurement time points (c) and (b) was 19.8 g (SD 22.6, median: 12.5) (3.9%).

Since heart weight is the most important parameter in the determination of cardiac hypertrophy in lifetime and heart weight should not exceed 500 g for men and 400 g for women as a reasonable limit (depending on age and/ or body surface area) [13, 14], we examined in how many cases the weight was measured as too high at the three time points. In men, heart weight exceeded 500 g in 18 decedents at measuring time point (a). In two of these cases, the heart weight was less than 500 g at the second measurement time point (b). Neither of these two cases fell below the heart weight of 500 g after rinsing and drying. In women, heart weight exceeded 400 g in 14 cases at measuring time point (a). Four of these cases fell below 400 g after dissection and two of them again after rinsing and drying.

### The difference in heart weights between measurement time points and between dissection methods

**Fig. 1:**
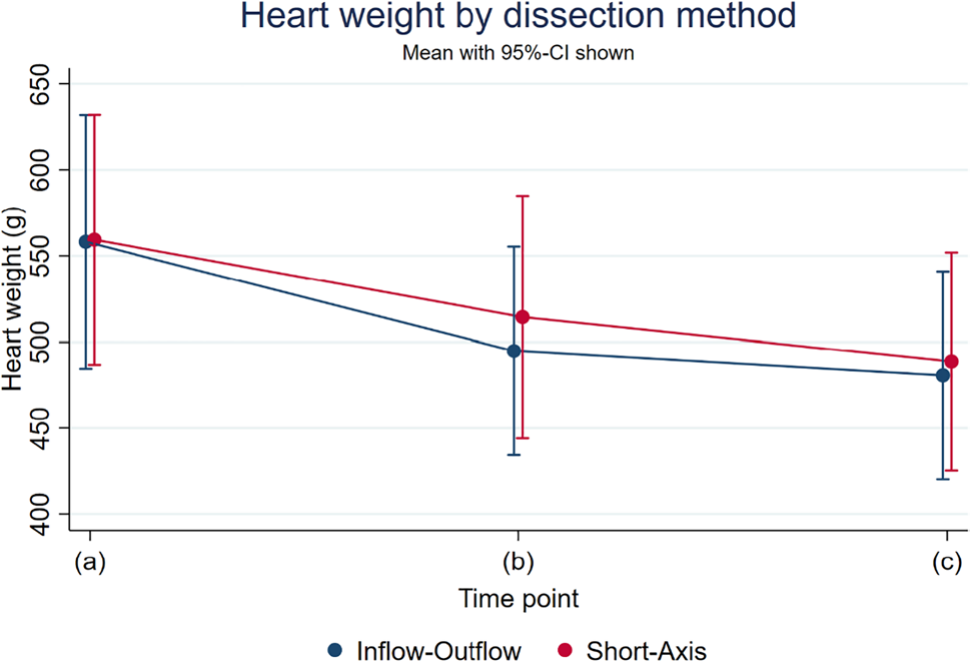
Changes in heart weight by dissection method. (**a**) After dissecting the heart out of the pericardial sac, (**b**) after complete dissection using the inflow-outflow or the short-axis method with manual removal of blood and blood clots, (**c**) after rinsing and drying

The baseline value is 558.8 g. After dissection and manual removal of blood, blood clots, and postmortem clots (b), the mean heart weight was 494.9 g with a median of 446.0 g (SD 169.9, range: 252.0–1026.0) using the inflow-outflow method and 514.5 g with a median of 454.0 g (SD 189.9, range: 242–895) using the short-axis method. After rinsing and drying (c), the heart weight was 480.7 g (SD 168.4, median: 432.5, range: 210.0–1016.0) using the inflow-outflow method and 488.7 g (SD 170.6, median: 442.5, range: 230–805) using the short-axis method. The model revealed an interaction between time point and dissection method (*p* = 0.041) (Fig. 1). Between time points (b) and (a), there was a decrease in heart weight of 64.8 g, 95%-CI [50.3;79.3] (11.6%; presented as adjusted mean values) after dissection using the inflow-outflow method and 43.3 g, 95%-CI [28.3;58.3] (7.8%) after short-axis dissection (difference between methods 21.5 g, 95%-CI [0.6;42.5], *p* = 0.044). At measurement time point (c), the heart weight was on average 79.0 g, 95%-CI [64.5;93.5] (14.1%) lower after using the inflow-outflow method and 69.1 g, 95%-CI [54.1;84.1]) (12.4%) lower after using the short-axis method compared to the measurement time point (a) (difference between methods less pronounced 9.9 g, 95%-CI [-11.0;30.9], *p* = 0.354). Using the short-axis dissection, the decrease in heart weight after rinsing and drying (compared to measurement time point (b)) was higher than using the inflow-outflow method (25.8 g, 95%-CI [17.8;33.8]; 5.1% vs. 14.2 g, 95%-CI [6.5;21.9]; 2.9%; difference between methods *p* = 0.041). Heart weight in women was on average overall 25 g lower (95%-CI [3.5;46.8]) compared to men (*p* = 0.023). The heart weights in this study increase with each BMI point at average by 1.6 g (95%-CI [-0.1;3.2], *p* = 0.063) and decrease with each year of life at average by 0.52 (95%-CI [-0.12;1.16], *p* = 0.109).

## Discussion

Cardiac hypertrophy can be a precipitant of arrhythmias and sudden cardiac death, and is frequently identified in forensic autopsies. Increased heart weight is often due to hypertrophy of the left ventricle, which can occur as a result of numerous causes, including hypertension, steroid abuse, valvular stenosis, amyloidosis, or obesity [15]. Especially in men, heart weight is commonly increased in cases with cardiac causes of death [16, 17]. The heart weight obtained at autopsy can be compared with previously established tables to determine if cardiac pathology is present [13, 14, 18]. In the diagnosis of sudden cardiac death, heart weight plays an important role in autopsy practice. However, in addition to a complete autopsy and the interpretation of all morphological changes, also histological, immunohistochemical, and toxicological analyses must be performed in order to make an appropriate diagnosis. For this reason, heart weight should be recorded as accurately as possible at autopsy. Blood, postmortem clots, and blood clots are not part of the heart weight and should be removed [5]. Heart weight, unlike ventricular wall thickness and volume, is a reliable measurement because it remains relatively constant after death [19] (provided decomposition changes are not present). While standardized methods exist for dissection of the heart [5, 12], no standard method exists for recording heart weight. Literature reports vary between weighing the heart after cutting off the cardiac apex without opening the chambers and after complete dissection [5, 8, 20], with Lee et al. [20] recommending reporting heart weight after complete dissection.

Following the recommendation of Garland et al. [10] who also used both dissection methods but weighed the heart at two different time points, this study documented the heart weight of 58 decedents at three different measurement time points using two standard dissection methods. After eviscerating/removing the heart out of the pericardial sac (a), the heart was dissected using either the inflow-outflow (*n* = 30) or the short-axis (*n* = 28) method, as recommended by the European and American guidelines [5, 12]. After dissection (b), and rinsing and drying (c), heart weight was lower than at the previous measurement time point with both dissection methods. The magnitude of weight differences between methods depended on the time point considered. At measurement time points (b) and (c) in comparison to (a), the heart weight was 11.6% (64.8 g) and 14.4% (79.0 g) lower using the inflow-outflow method, respectively, in comparison to short-axis dissection (7.8% (43.3 g); 12.4% (69.1 g) respectively). This could be explained by facilitated manual removal of blood and blood clots since the heart has a larger area in the dissected state than after the transversal slices sections in the short-axis method. In addition, it was shown that the weight loss after rinsing and drying (c) was higher after dissection using the short-axis method (5.1%; 25.8 g) in comparison to using the inflow-outflow method (2.8%; 14.2 g) (*p* = 0.041). Possibly after short-axis dissection, the heart is more easily rinsed and dried. In addition, mean heart weight was found to be 25 g lower in females than in males. This is not surprising, since heart weight is lower on average in women than in men [13, 14].

In contrast to the study by Garland et al. [10] this study showed a difference between the dissection methods depending on the measurement time point, whereby the study by Garland et al. referred to other measurement time points. The study by Loper et al. [11] also showed significant differences in heart weights at the three different time points with the lowest heart weight after dissection and pat drying.

The results suggest that the heart should not be weighed before dissection and manual removal of blood and blood clots because the heart weight is then overstated. We indicate that the measurement time point (c) is optimal regardless of the dissection method – although the weight loss between (b) and (c) was higher with the short axis method –, and we recommend that the heart be rinsed and dried with a surgical towel after preparation. Which of the two dissection methods is ultimately used is left to the discretion of the forensic medical examiner.

## Limitations

The deceased were mainly cardiology and oncology patients with a mean age of 72.2 years and these results cannot be applied to the pediatric population. This study refers exclusively to the recording of fresh non-formalin fixed heart weight at autopsy. No reference to clinical cause of death was made and thus the results are only relevant in autopsy practice. Because only a partial autopsy was performed in all cases, no association between heart weight and autoptic cause of death was determined, especially since this was beyond the scope of the paper. The purpose of this given study was not to establish a relationship between heart weight and cause of death. Since the aim of this study was to compare heart weights at the measurement time points, heart was weighed before the coronary arteries were opened and the great vessels were trimmed (c). If heart weight at autopsy is to be compared with standard values, it is recommended that the coronary arteries are opened and the great vessels trimmed before weighing. The high mean heart weight in this study likely reflects the high-risk population group (decedents over 18 years of age who had echocardiography up to six months before death) for cardiac pathology. Caution should therefore be taken in applying the findings of this study to the general autopsy population as mean differences between heart dissection methods prior to weighing may be greater or lesser in a population group with smaller heart weights on average.

## Data Availability

The datasets generated during and/or analysed during the current study are available from the corresponding author on reasonable request.
